# Intracellular sensing with transparent graphene-nanotube electrodes

**DOI:** 10.1088/2053-1583/ae4bec

**Published:** 2026-03-06

**Authors:** Xiao Fan, Jieun Park, Vaishali Malik, Lin Feng, Xin Zhang, Huilu Bao, Xiaoyu Zhang, Govindarajan Srimathveeravalli, Stephen S Nonnenmann, Jinglei Ping

**Affiliations:** 1 Department of Mechanical and Industrial Engineering, University of Massachusetts Amherst, Amherst, MA 01003, United States of America; 2 Institute of Applied Life Sciences, University of Massachusetts Amherst, Amherst, MA 01003, United States of America

**Keywords:** intracellular sensing, graphene, CNT, electroporation

## Abstract

Transparent electrodes based on graphene are ideal for multimodal cell sensing. However, the atomically flat basal plane of graphene limits the applications largely to capacitive detection of extracellular signals. Here, we develop a biocompatible graphene–carbon nanotube (CNT) hybrid (Bio-GCH), consisting of a graphene substrate covalently decorated with sparsely distributed CNT structures. Bio-GCH electrodes enable low-bias (~4 V) nano-electroporation of cardiomyocytes and high-quality intracellular recordings, with CNTs serving as the dominant electrical transduction channel. At the same time, Bio-GCH electrodes retain the key advantages of graphene electrodes, including high optical transparency, electrical mobility, electrochemical stability, and biocompatibility.

## 1. Introduction

Transparent electrodes are critical for cell interfacing as they enable multimodal sensing through electrical and optical techniques [1]. Graphene has emerged as an ideal material for transparent electrodes due to its high biocompatibility, optical transparency, carrier mobility, electrochemical stability, corrosion resistance, and mechanical flexibility [2–4]. Unlike indium tin oxide (ITO), which is brittle and degrades under mechanical stress or in aqueous environments, graphene combines outstanding mechanical flexibility with chemical stability, making it suitable for long-term biointerfaces [5]. Compared with ultrathin metals or nanomeshes, graphene provides higher stability, optical transparency, and conductivity, enabling seamless integration with optical imaging and high-fidelity electrical recordings. Conductive polymers such as PEDOT:PSS, although biocompatible, often suffer from limited long-term stability and reproducibility [6], issues not encountered with graphene.

**Figure 1.**
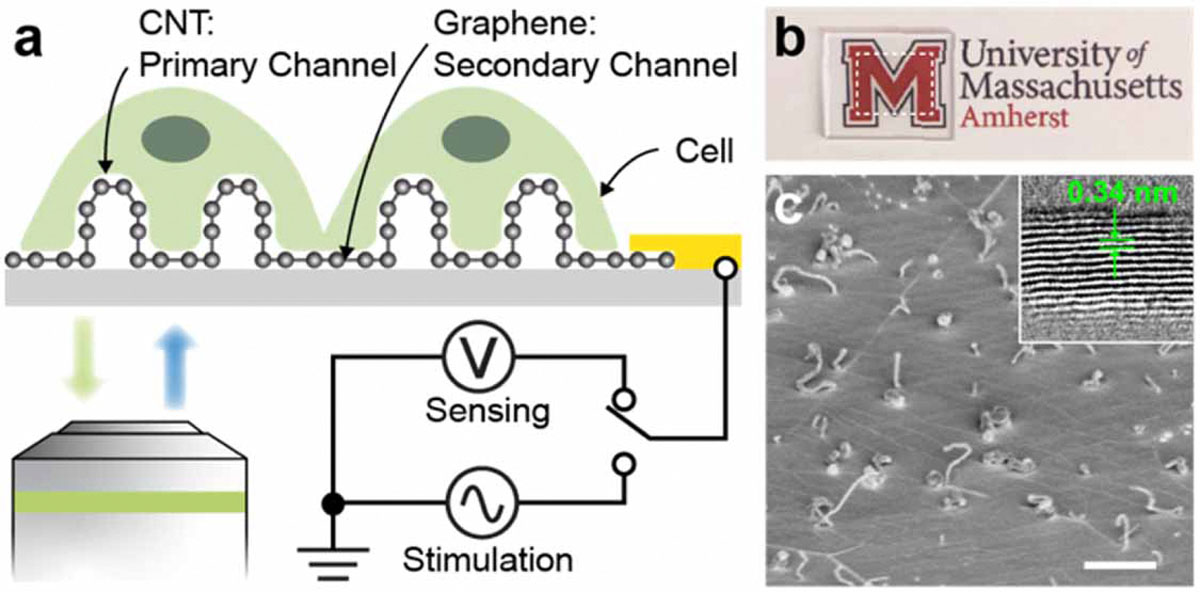
Transparent Bio-GCH electrode for cell interfacing. (a) Schematic of the Bio-GCH electrode setup for cell stimulation and signal detection. (b) Photograph of a Bio-GCH sample (dashed box) transferred onto a glass substrate. (c) Scanning electron microscopy (SEM) image of Bio-GCH; inset: transmission electron microscopy (TEM) image showing the multiwalled structure of CNTs. Scale bar: 1 *μ*m.

Despite these advantages, the atomically flat basal plane of graphene hinders intracellular access, restricting its use largely to capacitive sensing in transistor channels. Moreover, the high interfacial impedance of graphene electrodes substantially degrades signal quality [7, 8]. To overcome these limitations, we developed transparent electrodes based on a biocompatible graphene–carbon nanotube (CNT) hybrid (Bio-GCH) prepared via chemical vapor deposition (CVD), enabling intracellular biosensing as shown in figure 1(a).

## 2. Results

Unlike earlier GCHs, which suffer from low transparency due to densely packed CNTs grown with film catalysts and relatively high precursor flow rates [9–14], (supplementary information table S1) our Bio-GCH features a low CNT density and maintains 93.9% optical transmittance—comparable to that of bare CVD-grown graphene (98%) [15]. This is enabled by an optimized CVD process that employs iron-oxide nanoparticle (NP) catalysts and low ethylene precursor flow rates, implemented through four sequential steps: monolayer graphene synthesis, deposition of sparse Fe_3_O_4_ NPs with optimized areal density (~2.2 ± 0.2 *μ*m^−2^), NP annealing, and CNT growth on the substrate graphene under low precursor flow (typically 1 sccm). Within 20 min, this process yields vertically grown CNTs (figure 1(c)) with lengths of 990 ± 120 nm and an average inter-CNT spacing of 0.7 *μ*m—suitable for probing and spatially resolving typical live cells. Transmission electron microscopy (TEM) indicates their multi-walled structure (figure 1(c), inset), with diameters of 43 ± 13 nm, closely matching those of the annealed NPs (43 ± 10 nm; supplementary information figure S1). In contrast to CNTs synthesized by earlier methods [9–14] (<20 nm in diameter), these large diameters provide greater mechanical stiffness and buckling resistance, thereby enhancing stability during transfer, device fabrication, and biointerfacing with minimal deformation or fracture [16].

**Figure 2.**
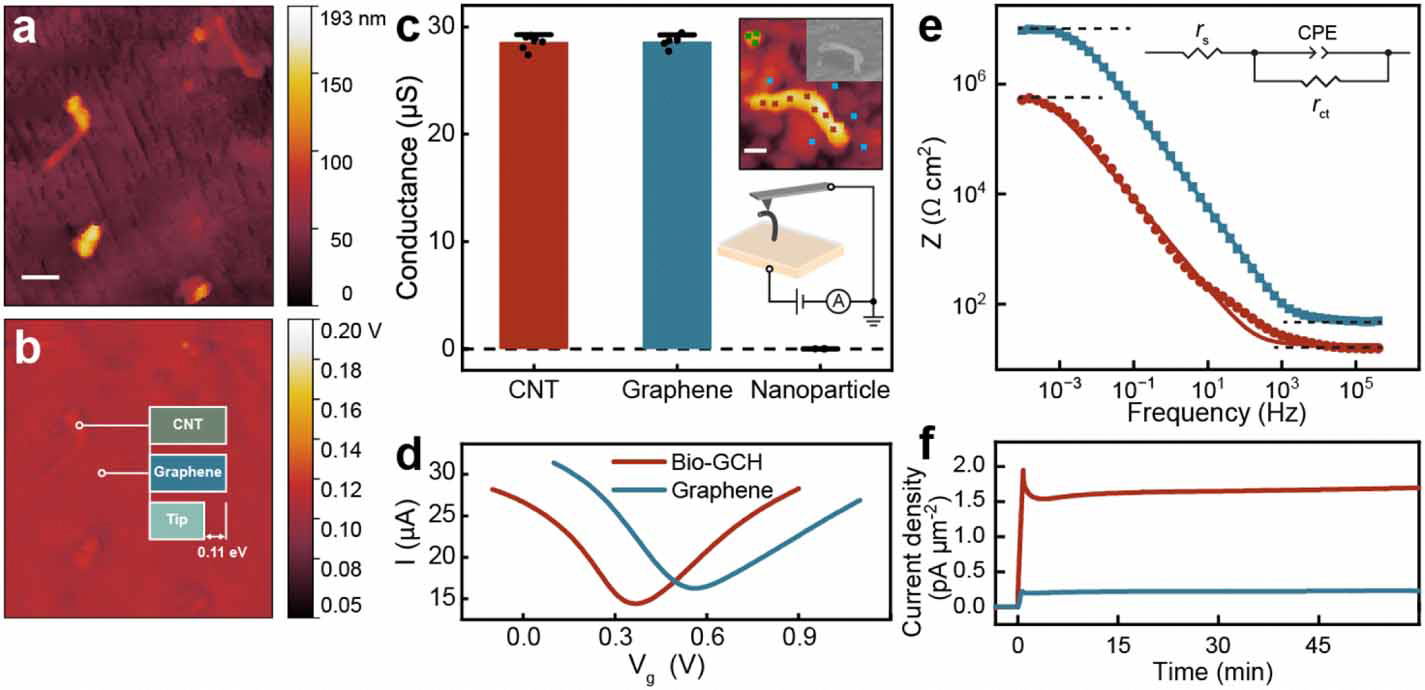
Characterization of Bio-GCH and Bio-GCH electrodes. (a) AFM image of the Bio-GCH surface morphology. Scale bar: 500 nm. (b) KPFM map of the region in (a). Inset: schematic illustrating the work-function difference between the AFM tip and two regions: CNT and underlying graphene. (c) Conductance values measured at distinct Bio-GCH locations using conductive AFM. The dashed line serves as a guide for the eye. Top inset: AFM image with measurement locations (CNT: red, substrate graphene: blue, nanoparticle: green) and a 60°-tilted grayscale SEM image of approximately the same region. Scale bar in the AFM image: 100 nm. Bottom inset: schematic of the conductive AFM measurement configuration. (d) Current versus liquid-gate voltage (*I*–*V*_g_) characteristics for bare CVD graphene and Bio-GCH field-effect transistors (FETs). (e) Bode plot of impedance magnitude for graphene and Bio-GCH electrodes. Solid curves represent fits using a simplified Randles circuit model (inset). Horizontal dashed lines highlight plateaus corresponding to *r*_s_ (at high frequencies) and *r*_ct_ (at low frequencies). (f) Time-dependent current density for graphene–graphene and Bio-GCH–Bio-GCH electrode pairs after applying a 2-V bias at *t* = 0. Color coding for bare graphene and Bio-GCH is consistent across panels (d)–(f).

The vertically grown CNTs are covalently bonded to substrate graphene [14]. Kelvin probe force microscopy (KPFM; figures 2(a) and (b)) shows a minimal work-function difference between CNTs and substrate graphene, indicating a shared Fermi level. Conductive atomic force microscopy (AFM; figure 2(c)) further demonstrates identical interfacial resistances between the AFM tip and substrate graphene (34.9 ± 0.8 kΩ) and between the tip and CNTs (35.0 ± 0.8 kΩ), confirming minimal graphene-CNT contact resistance.

Enhancements in interfacial conductivity typically induce charge-carrier scattering, thereby degrading conductivity [17]. Current–gate voltage (*I*–*V*_g_) measurements (figure 2(d)) show that BioGCH retains carrier mobility comparable to bare CVD graphene and exhibits reduced hole doping, a favorable feature for electronic applications. This mobility retention arises from the low CNT areal density (2.2 ± 0.2 *μ*m^−2^) offered by our method, which generates significantly less scattering than that caused by defects in graphene (defect density: ~2 × 10^3^ *μ*m^−2^, estimated from its mobility of ~2 × 10^3^ cm^2^ V^−1^ s^−^[1, 18, 19].

Electrochemical impedance spectroscopy (EIS) was conducted to assess the performance of Bio-GCH electrodes. The impedance amplitude–frequency spectra (figure 2(e)) show two characteristic plateaus for both bare graphene and Bio-GCH, corresponding to solution resistivity (*r*_s_) and charge-transfer resistivity (*r*_ct_). These data are well fitted by a simplified Randles model (figure 2(e), inset). Based on this model, the Bio-GCH electrode exhibits *r*_ct_ = 0.62 ± 0.01 MΩ cm^2^, corresponding to a charge-transfer conductivity (*σ*_ct_) of 1.61 ± 0.03 *μ*S cm^−2^, and an effective areal capacitance (*c*_*i*_) of 473 ± 2 *μ*F cm^−2^. Relative to the bare-graphene electrode, the Bio-GCH electrode shows a 16-fold and 83-fold enhancements in *σ*_ct_ and *c*_*i*_, respectively. The EIS results are consistent across devices, with fluctuations determined by the 10% uncertainty in NP density.

Notably, Bio-GCH electrodes retain the electrochemical stability of bare graphene, owing to the strong C–C covalent bonds anchoring CTNs to the substrate graphene [14]. Under a 2 V bias (sufficient to induce electrochemical effects such as pH-gradient formation) [20], the current density between two Bio-GCH electrodes stabilizes at 1.67 pA *μ*m^−2^ and remains constant for hours (figure 2(f)). This value is 8× higher than that of bare graphene electrodes (0.22 pA *μ*m^−2^) under identical bias conditions, yet without compromising stability. Such robustness is essential for deploying Bio-GCH electrodes in bioelectrical stimulation applications.

**Figure 3.**
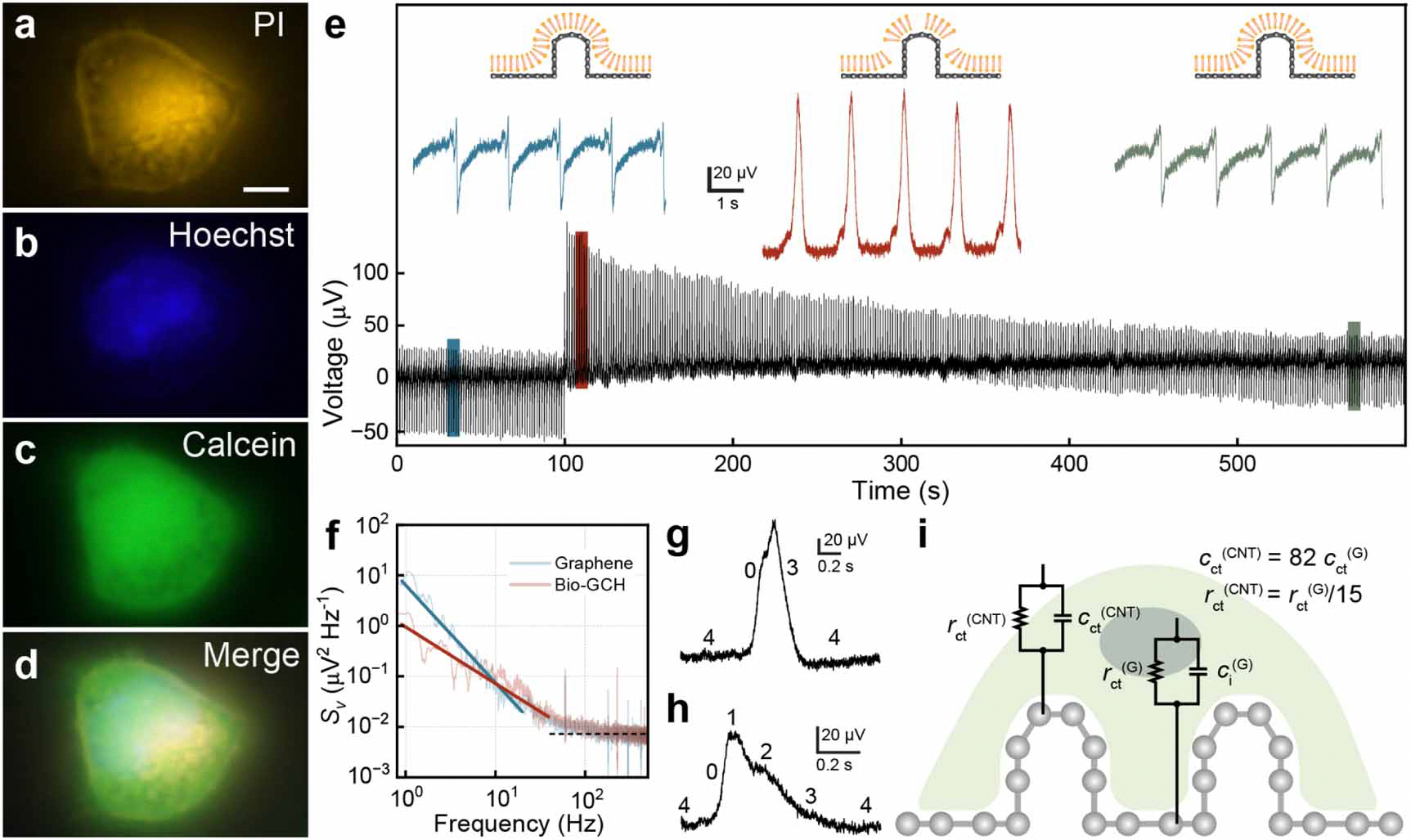
Electrophysiological recordings of HL-1 cells using Bio-GCH electrodes. (a)–(d) Fluorescence microscopy validation of HL-1 cell viability following nano-electroporation. Scale bar: 5 *μ*m (applies to all images). (e) Action potential measurements before and after nano-electroporation (triggered at *t* = 100 s). Representative action potentials (color-shaded regions) are magnified above the full-range trace, with data colors matching the corresponding regions. Top: schematics illustrating pore formation and sealing. (f) Noise power spectral density (*S*_*v*_) of Bio-GCH and bare graphene electrodes during extracellular recordings. Dark lines denote linear fits to the data (lighter-colored lines); the dashed line indicates the thermal noise floor. (g) Intracellular action potential of pacemaker HL-1 cells. (f) Intracellular action potential of non-pacemaker HL-1 cells. Numerical labels in (g) and (h) indicate distinct phases within a cycle. (i) Equivalent electrical circuit diagram modeling the Bio-GCH electrode interface. Parameter values are derived from electrochemical impedance spectroscopy measurements.

The high curvature of CNTs amplifies the local electric field by ~10^2^-fold, [21, 22] enabling nano-electroporation of cells cultured on Bio-GCH electrodes at low voltages. When 4 V, 200 *μ*s biphasic pulses (40 pulses over 2 s) are applied, transient pore formation is induced without compromising cardiomyocyte viability (intracellular recording success rate: >90%) (figures 3(a)–(d); supplementary information figure S2, table S2). In contrast, bare CVD graphene electrodes fail to induce electroporation even at 8 V (Supplementary Information figure S3). Prior to electroporation, Bio-GCH electrodes readily record biphasic extracellular action potentials from HL-1 cardiomyocytes cultured on the electrodes (figure 3(e), pre-electroporation trace at 100 s). The noise level of extracellular recording on Bio-GCH is markedly lower than that on baregraphene (supplementary information figure S4), yielding a 3.6-fold improvement in signal-to-noise ratio. This enhancement is consistent with the 16-fold reduction in $r_{ct}$ for Bio-GCH electrodes, which lowers Johnson noise by fourfold, following the relationship $v_{\Delta f}∼\sqrt{R_{ct}}$, where $v_{\Delta f}$ represents the voltage noise per unit bandwidth.

Power spectral density analysis of extracellular action potential noise (figure 3(f)) indicates a high-frequency (>300 Hz) plateau of 0.007 ± 0.002 *μ*V^2^ Hz^−1^ for both Bio-GCH and bare graphene electrodes, reflecting Johnson noise from a resistivity of 22 ± 6 Ω cm^2^. This agrees well with the *r*_s_ of Bio-GCH measured by EIS (18.0 ± 0.1 Ω cm^2^; figure 2(e)). At lower frequencies, Bio-GCH electrodes exhibit 1/*f* noise (slope = −1.09 ± 0.01), characteristic of diffusion-dominated, non-polarized transduction resulting from low interfacial voltage due to reduced impedance. In contrast, bare graphene displays 1/*f*^2^ noise (slope = −1.93 ± 0.01), typical of drift-dominated, polarized interfaces [23]. These distinct noise signatures highlight that Bio-GCH electrodes enhance electrical cell interfacing by shifting the transduction mechanism from polarized to non-polarized behavior relative to bare graphene.

Post-electroporation, Bio-GCH electrodes record clear intracellular action potentials (figure 3(e), trace at 100 s), reflecting cytoplasmic activity. Within 400 s, signals revert to extracellular action potentials, indicating pore closure and membrane recovery. During the pore-open window, CNTs of BioGCH electrodes enable intracellular interrogation and precise analysis of cell-specific electrophysiology. The Bio-GCH electrodes can be reused for multiple intracellular access events with minimal degradation. Pacemaker cells exhibit triphasic intracellular potentials (figure 3(g)): Phase 4 slow depolarization (Na^+^ influx), Phase 0 rapid depolarization (Ca^2+^ influx), and Phase 3 repolarization (K^+^ efflux). In contrast, non-pacemaker cardiomyocytes display five distinct phases (figure 3(h)): Phase 0 rapid depolarization (Na^+^ influx), Phase 1 repolarization (K^+^ efflux), Phase 2 plateau (Ca^2+^/K^+^ currents), Phase 3 restoration to the resting potential (K^+^ efflux), and Phase 4 maintenance of a stable resting potential.

The predominance of cytoplasmatic signals after electroporation arises from the CNT transduction channel prevailing over the substrate-graphene channel in signal transduction. This can be quantified by modeling the Bio-GCH interface as a parallel circuit (figure 3(i)) comprising four components: the charge-transfer resistivity ($r_{ct}^{\left(CNT\right)}$) and areal capacitance ($c_{i}^{\left(CNT\right)}$) of CNTs, and the corresponding parameters for the graphene substrate $\left(r_{ct}^{\left(G\right)},c_{i}^{\left(G\right)}\right)$. The graphene-substrate values are comparable to those of bare CVD graphene, *i*.*e*. $r_{ct}^{\left(G\right)}=9.8\pm 0.1\;M\Omega \;{cm}^{2}$, $c_{i}^{\left(G\right)}=5.70\pm 0.01\;\mu F{\;cm}^{-2}$. From the EIS results of Bio-GCH electrodes, CNT-channel values are derived as $r_{ct}^{\left(CNT\right)}=0.66\pm 0.01\;M\Omega {\;cm}^{2}$ and $c_{i}^{\left(CNT\right)}=467\pm 2\;\mu F{\;cm}^{-2}$, corresponding to a 15-fold lower resistivity and an 82-fold higher areal capacitance relative to graphene substrate. Consequently, once CNTs penetrate cell membrane, they dominate signal transduction and enable high-fidelity intracellular action-potential recordings.

## 3. Conclusions

Bio-GCH electrodes retain advantageous properties of graphene while enabling intracellular sensing. The dual composition of CNTs and graphene in Bio-GCH further supports diverse functionalization and doping strategies, establishing a highly adaptable platform for high-specificity biosensing and stimulation [24]. Collectively, these attributes position Bio-GCH electrodes as a promising platform for advancing bioelectronic applications in cell development and tissue engineering [25].

From a manufacturing perspective, we present a simple, scalable CVD-based approach for anchoring nanostructures on planar substrates. By contrast, prior studies typically rely on complex fabrication techniques (*e.g.* focused ion beam) [26–28] that often require elaborate substrate passivation steps. The high stiffness and fracture strength of CNTs [16], despite their nanoscale diameters (sub-50 nm in this work), underpin the mechanical robustness of Bio-GCH, enabling reliable fabrication and stable performance in cell interfacing applications.

Our work also establishes a strategy to reduce the interfacial impedance of graphene electrodes without compromising their intrinsic properties. Bio-GCH electrodes exhibit interfacial impedance orders of magnitude lower than bare graphene, with CNTs serving as the primary transduction channel, while preserving graphene’s key attributes of high optical transparency, carrier mobility, and electrochemical stability. In contrast, existing impedance-reduction strategies—including noncovalent binding (*e.g. π*–*π* stacking [29–31] or physical adsorption [32, 33]) and covalent functionalization (*e.g.* dopant integration) [34–36]—often diminish graphene’s biocompatibility, transparency, or carrier mobility, and may result in unstable hybrids.

## 4. Methods

### Graphene and Bio-GCH synthesis

A copper foil (Alfa Aesar, 46986) was first treated with acetic acid for 10 min, loaded into a quartz tube (1 inch inner diameter, 4-foot length), and annealed at 1060 °C for 30 min under a mixed gas flow of hydrogen (99.999%, 200 sccm) and argon (99.999%, 500 sccm) to remove surface oxides. Monolayer graphene was then synthesized on the copper substrate via CVD at 1035 °C for 7 min using a gas mixture of hydrogen (99.999%, 9 sccm), argon (99.999%, 500 sccm), and methane (99.99%, 0.5 sccm).

For Bio-GCH growth, the graphene-coated copper foil was immersed in 1 mM ethanol (Fisher Scientific, BP2818500) solution of Fe_3_O_4_ NPs (Ocean NanoTech, SC0053; 60–70 nm diameter) for 10 min. After NP deposition, the sample was dried, transferred into a quartz tube, and annealed at 750 °C for 20 min under hydrogen (99.999%, 160 sccm) and argon (99.999%, 200 sccm) to reduce Fe_3_O_4_ NPs to Fe NPs. Ethylene (99.99%, 1 sccm) was subsequently introduced to initiate Bio-GCH growth.

### Force microscopy

AFM, conductive AFM, and KPFM were performed under ambient conditions using a Cypher ES atomic force microscope (Oxford instruments, Asylum Research). A conductive AC240TM-R3 probe (Oxford instruments; frequency = 70 kHz, stiffness = 2 N m^−1^) was used to scan regions of interest pre-identified via scanning electron microscopy (SEM).

In AFM and conductive AFM, topography was first mapped in contact mode at a scan rate of 0.7 Hz. Selected points on the scanned area were then subjected to current–voltage measurements by holding the tip in contact while applying a bias from—0.1 V to +0.1 V. A constant probe deflection setpoint (0.03 V) ensured consistent tip-sample contact.

For KPFM, a delta height (vertical offset between the probe and the sample surface) of 10 nm was maintained to ensure precise surface potential measurements. This distance effectively separated the topographical and electrostatic signals while minimizing mechanical interference. KPFM images were acquired using AC bias (*V*_AC_ = 3 V) with a scan rate of 0.5 Hz.

### Electron microscopy

Bio-GCH samples on copper substrates were imaged using an FEI Magellan 400 XHR-SEM at an accelerating voltage of 1 kV and a beam current of 25 pA. The average CNT length was determined by quantifying the ten longest CNTs within a 100 *μ*m^2^ area from SEM images.

For TEM analysis, a Bio-GCH sample or a graphene sample with annealed NPs was transferred to Quantifoil grids (Electron Microscopy Sciences, Q225-CR1.3). The multi-walled structure of the CNTs in the Bio-GCH was characterized using a JEOL JEM-2200FS TEM at an accelerating voltage of 200 kV. Imaging was also performed with a FEI Tecnai T12 TEM at an accelerating voltage of 120 kV. CNT and NP diameters were quantified using Fiji software.

For SEM imaging of HL-1 cells on the Bio-GCH, cells were fixed with 4% paraformaldehyde (Fisher Scientific, 23–305510) for 20 min, dehydrated in a graded ethanol series (30%, 50%, 70%, 90%, and three rinses with 100% ethanol), and treated with hexamethyldisilazane (HDMS; Thermo Scientific, 430850010) in ethanol at increasing concentrations (20%, 40%, 60%, 80%, and three immersions in 100% HDMS). A 5 nm gold layer was sputtered onto the samples prior to imaging with an FEI Magellan 400 XHR-SEM at an accelerating voltage of 5 kV and a beam current of 50 pA.

### Optical transmittance measurement

Samples transferred on glass substrates (Fisherbrand, 12543D; 200 *μ*m thickness) were analyzed using a Zeiss Observer 7 transmission optical microscope equipped with an Axiocam 702 mono CMOS camera. Transmittance was calculated as the ratio of light intensity transmitted through the sample on the glass substrate to that through the bare glass substrate, under identical white-light illumination conditions.

### Graphene/Bio-GCH electrode fabrication

A bare CVD graphene electrode was fabricated by spin-coating a 400 nm-thick poly(methyl methacrylate) (PMMA; Kayaku Advanced Materials, 950 PMMA A4) layer onto a graphene-copper film, followed by baking at 100 °C for 30 s. The copper film was etched away using a copper etchant (Alfa Aesar, 44 583). The resulting PMMA-graphene film was rinsed sequentially in three deionized (DI) water baths and transferred onto a glass substrate prefabricated with 65 nm-thick Cr/Au contacts (deposited via photolithography and e-beam evaporation). After drying overnight at room temperature, the chip was soaked in acetone (Fisher Chemical, A18P-4) for 3 h to dissolve the PMMA, rinsed with isopropyl alcohol (IPA; Fisher Chemical, A416P-4), and dried under a nitrogen stream.

For fabricating Bio-GCH electrodes, a PMMA mixture was prepared by blending 90% (v/v) of a 16% (w/w) PMMA (Sigma-Aldrich, 81 489; molecular weight ~2000) in anisole (Fisher Scientific, 153922500) with 10% (v/v) of 950 PMMA A4 (Kayaku Advanced Materials). This mixture was spin-coated onto Bio-GCH on a copper substrate and dried overnight. The copper substrate was etched with a copper etchant (Alfa Aesar, 44 583), and the PMMA-Bio-GCH film was rinsed sequentially in three DI water baths before being transferred onto a glass substrate with prefabricated 65 nm-thick Cr/Au contacts. After drying overnight, the chip was soaked in acetone (Fisher Chemical, A18P-4) for 3 h to remove PMMA. It was then immersed in hexamethyldisilazane (HMDS; Fisher Scientific, 430850010) solutions at increasing concentrations (20%, 40%, 60%, 80% v/v in acetone), followed by three rinses in pure HMDS. Finally, the sample was air-dried at room temperature.

### Graphene/Bio-GCH transistor fabrication

A graphene/Bio-GCH channel was fabricated by transferring the graphene/Bio-GCH onto a silicon wafer with a 500 *μ*m-thick thermal silicon dioxide layer (University Wafer, 1432) and prefabricated Cr/Au contacts (65 nm-thick). The channel was defined via photolithography using a bilayer photoresist (S1813 layered over PMGI SF 2S; Kayaku Advanced Materials). Unwanted graphene/Bio-GCH regions were removed by oxygen plasma etching (40 W, 4 mTorr, 1 min). Residual photoresist was stripped using Remover PG (Kayaku Advanced Materials) at 60 °C for 30 min, followed by IPA (Fisher Chemical, A416P-4) rinsing and drying with a nitrogen stream. SU-8 2002 photoresist was then applied to passivate non-channel regions.

### Device assembly

A polypropylene well was attached to a fabricated electrode or transistor chip using spacer tape (Adhesives Research, 94 119), which defined an exposed testing area of 50 *μ*m × 100 *μ*m for the electrode or 50 *μ*m × 120 *μ*m for the transistor.

### Electron transport measurement

The graphene/Bio-GCH transistor channel was immersed in 1× physiological buffer solution (PBS; Fisher Scientific, BP661–50; pH 7.4, 150 mM ionic strength). A 0.25 mm-diameter Pt wire served as the liquid gate electrode, controlled by a Keysight 2987B electrometer that was also used to measure the drain-source current. The source-drain voltage was applied using a Keithley 2450 Sourcemeter.

### Electrochemical measurement

EIS were performed in 1× PBS (Fisher Scientific, BP661–50; pH 7.4, ionic strength 150 mM) using a Gamry Reference 600 High-Performance Potentiostat/Galvanostat/ZRA system. A three-electrode setup were employed with an Ag/AgCl reference electrode and a Pt plate counter electrode, using the graphene/Bio-GCH electrode as the working electrode. EIS measurements were acquired with a 10 mV AC amplitude.

### HL-1 cell culture

HL-1 cardiac muscle cells (Sigma-Aldrich, SCC065; ⩽10 passages) were cultured in T-25 flasks with HL-1 expansion medium. The medium consisted of 87% Claycomb basal medium (Sigma-Aldrich, 51 800 C), 10% fetal bovine serum (Sigma-Aldrich, TMS-016), 0.1 mM norepinephrine (Sigma-Aldrich, A0937), 2 mM L-glutamine (Sigma-Aldrich, TMS-002), and 100–Uml^−1^ penicillin-streptomycin(Sigma-Aldrich, TMS-AB2). At 100% confluency, cells were loaded into the device’s well at a density of ~3500 cells mm^−2^ and maintained in an incubator (37 °C, 5% CO_2_). Culture medium was replaced daily.

### Cell recording and electroporation

HL-1 action potentials were recorded at 1 kHz using a Bio-GCH electrode connected to a Keysight 2987B electrometer, with an Ag/AgCl reference electrode.

For nano-electroporation, a biphasic pulse train (4 V, 200 *μ*s pulse width, 20 Hz, 2 s) was delivered through a Bio-GCH microelectrode.

### Fluorescence microscopy

To assess electroporation efficiency, 500 *μ*L of Tyrode’s solution (Alfa Aesar, J67593; pH 8.1) supplemented with 5 *μ*g ml^−1^ propidium iodide (PI; Thermo Scientific, AAJ66584AB) was added to the polypropylene well of an electrode or transistor chip pre-seeded with cultured cells and incubated at 37 °C for 2 min. After electroporation, the chip was returned to the incubator (37 °C, 5% CO_2_) for 10 min to allow PI uptake. The PI solution was aspirated, and the chip was washed twice with Dulbecco’s phosphate-buffered saline (DPBS; Thermo Scientific, 14 190 144).

The PI-stained cells were additionally stained with 500 *μ*l of DPBS containing 2 *μ*g ml^−1^ Calcein AM (Invitrogen, C3099) and 2 *μ*g ml^−1^ Hoechst (Invitrogen, H3570) for 30 min. After incubation, the staining solution was aspirated, and the chip was washed twice with DPBS within the well. Finally, 500 *μ*l of Tyrode’s solution was added to the well for imaging.

Fluorescence imaging was performed using a Nikon Ti2 microscope equipped with a Prime 95B camera and a 60× oil immersion objective. Excitation wavelengths were set to 365 nm (Hoechst), 488 nm (Calcein AM), and 561 nm (PI), with corresponding band-pass filters of 414–450 nm, 500–530 nm, and 580–610 nm, respectively.

## Supplementary Material

Supplementary Information

Supplementary Information available at https://doi.org/10.1088/2053-1583/ae4bec/data1.

## Data Availability

All data that support the findings of this study are included within the article (and any supplementary files).
