# Supplementary material for: Intracellular sensing with transparent graphene-nanotube electrodes
Source: 2d Mater. Author manuscript; Available in PMC 2026 Jul 21. (PMC13384486; doi:10.1088/2053-1583/ae4bec)
Supplement: Supplementary Information [file NIHMS2184707-supplement-Supplementary_Information.pdf]

Supplementary Information

Intracellular Sensing with Transparent Graphene-Nanotube Electrodes

*Xiao Fan,[1] Jieun Park,[1] Vaishali Malik,[1] Lin Feng, [1] Xin Zhang,[1] Huilu Bao,[1] Xiaoyu Zhang,[1] Govindarajan Srimathveeravalli,[1,2] Stephen S. Nonnenmann,[1] Jinglei Ping[1,2,]**

[1]Department of Mechanical and Industrial Engineering, University of Massachusetts Amherst, Amherst, MA 01003, USA

[2]Institute of Applied Life Sciences, University of Massachusetts Amherst, Amherst, MA 01003, USA

*Corresponding author: Jinglei Ping

Email: ping@engin.umass.edu

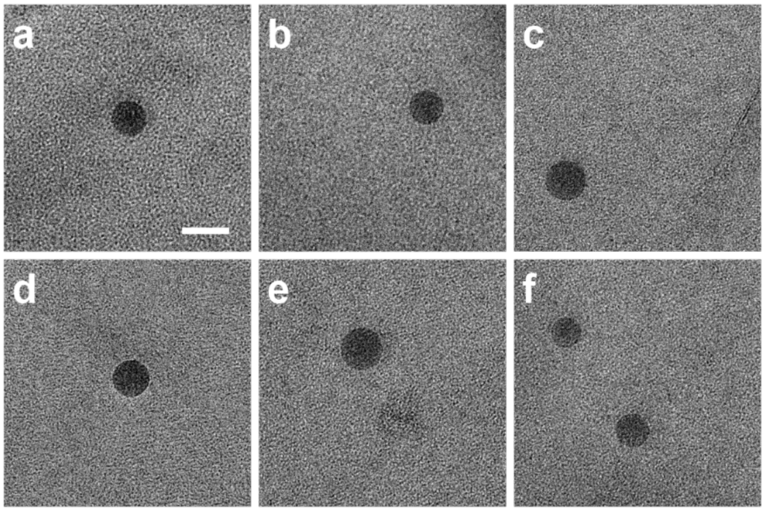

Figure S1. Representative TEM images of annealed catalytic NPs. Scale bar: 50 nm (applied to all images).

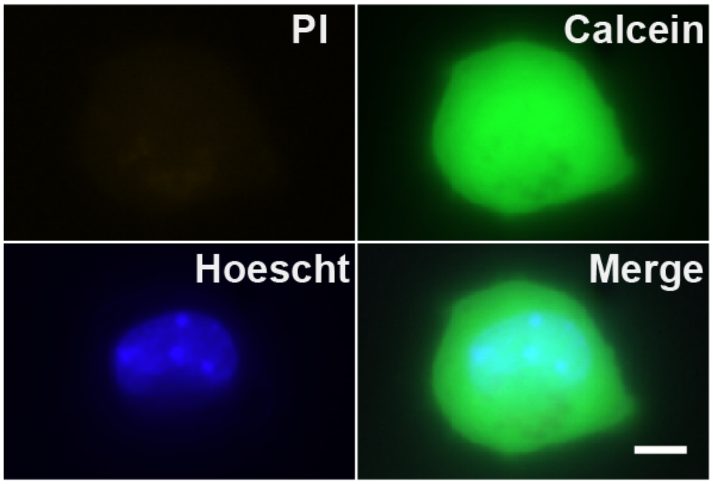

Figure S2. Control experiments showing no PI entry into cells without electroporation on a Bio-GCH electrode. Scale bar: 5 µm (applies to all images).

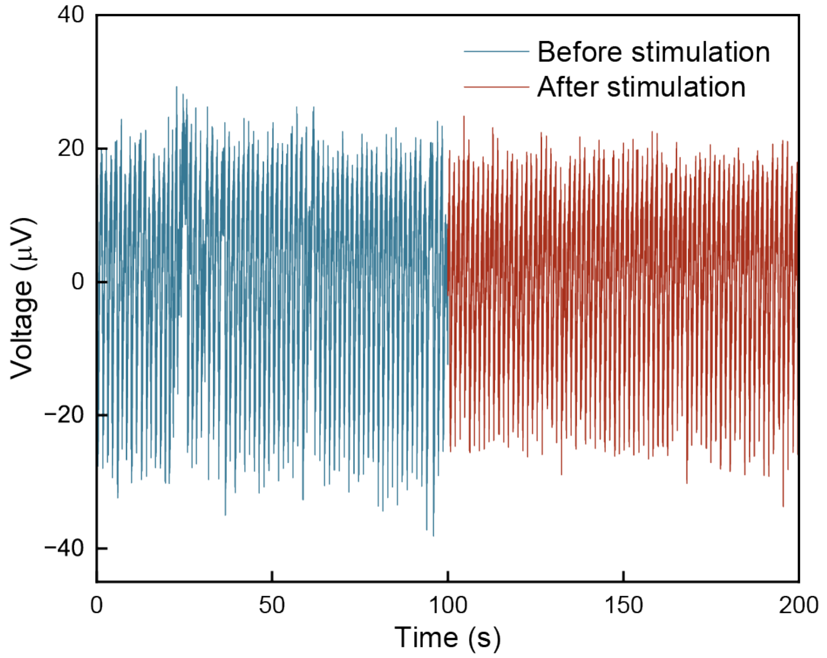

Figure S3. Action potentials recorded using a bare-graphene electrode. HL-1 cells on the microelectrode are stimulated with an 8-V pulsatile train. Post-stimulation action potentials retain their extracellular waveform.

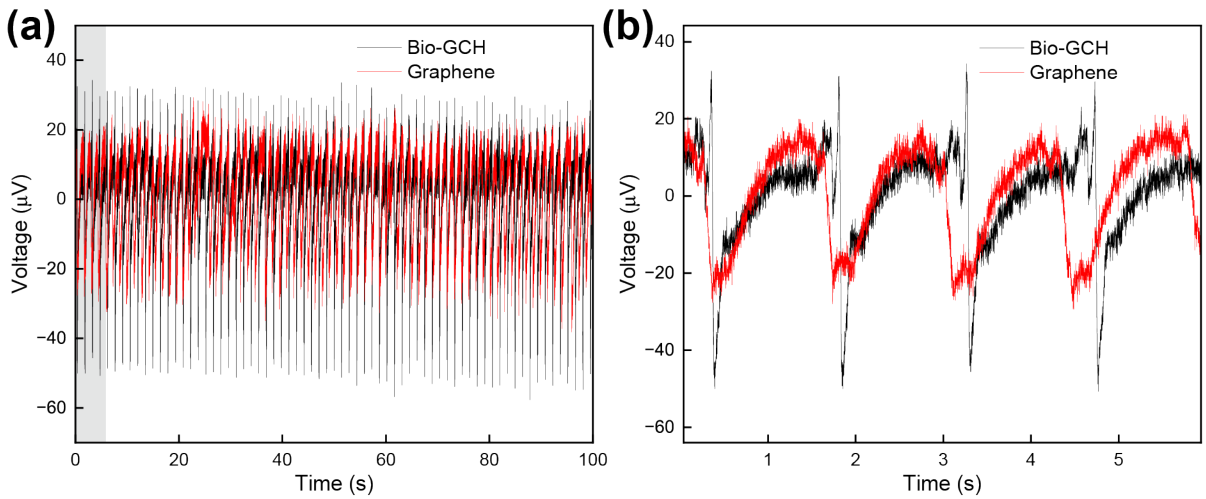

Figure S4. Extracellular action potential recordings comparing Bio-GCH and bare CVD graphene electrodes. a) Recordings over a 100-s period. b) Enlarged view of the shaded region in (a), showing action-potential signals during a 6-s interval.

Table S1. Comparison of Graphene–CNT Hybrid Structures

	CNT Density	Transparency	Electrochemical Data	Application Focus
Kondo et al. (2008)[1]	Dense vertical MWCNTs	Not reported	Not reported	Electronics
Paul et al. (2010)[2]	Moderate vertical density	Not reported	Not reported	Energy storage concept
Kim et al. (2012)[3]	Dense vertical SWCNTs	Not reported	653.7 µF/cm² areal capacitance	Supercapacitor
Zhao et al. (2012)[4]	Dense vertical SWCNTs	Not reported	Improved Li–S rate capacity (~1140 mAh/g at 0.2C)	Battery cathode
Zhu et al. (2012)[5]	Dense vertical SWCNTs	Opaque	~2000 m²/g surface area	Supercapacitor
Wang et al. (2014)[6]	High-density VACNTs	Opaque	900 mAh/g, stable over 250 cycles	Battery anode
This work (GCH)	Sparse vertical MWCNTs	~94%	>16× lower impedance than graphene	Transparent, low-impedance biointerface

Table S2. Functions and properties of fluorophore dyes used for verification of cell electroporation

Fluorophore Dyes	Entry into Cells	Fluorescence Mechanism	Usage
PI (Propidium Iodide)	Does not permeate the membranes of live cells; enters dead cells with compromised membrane integrity	Binds to nucleic acids and emits red fluorescence	Verification of electroporation or identification of dead cells
Calcein AM	Easily penetrates the membranes of live cells.	Once hydrolyzed by intracellular esterases, it becomes a fluorescent molecule emitting green fluorescence	Labelling and detecting live cells
Hoechst	Penetrates the membranes of both live and dead cells	Binds to DNA and emits blue fluorescence	Staining cell nuclei to observe nuclear morphology

Reference

1. Kondo, D.; Sato, S.; Awano, Y., Self-organization of novel carbon composite structure: graphene multi-layers combined perpendicularly with aligned carbon nanotubes. *Applied physics express* **2008,** *1* (7), 074003.

2. Paul, R. K.; Ghazinejad, M.; Penchev, M.; Lin, J.; Ozkan, M.; Ozkan, C. S., Synthesis of a pillared graphene nanostructure: a counterpart of three-dimensional carbon architectures. *Small* **2010,** *6* (20), 2309-2313.

3. Kim, Y.-S.; Kumar, K.; Fisher, F. T.; Yang, E.-H., Out-of-plane growth of CNTs on graphene for supercapacitor applications. *Nanotechnology* **2012,** *23* (1), 015301.

4. Zhao, M.-Q.; Liu, X.-F.; Zhang, Q.; Tian, G.-L.; Huang, J.-Q.; Zhu, W.; Wei, F., Graphene/single-walled carbon nanotube hybrids: one-step catalytic growth and applications for high-rate Li–S batteries. *ACS nano* **2012,** *6* (12), 10759-10769.

5. Zhu, Y.; Li, L.; Zhang, C.; Casillas, G.; Sun, Z.; Yan, Z.; Ruan, G.; Peng, Z.; Raji, A.-R. O.; Kittrell, C., A seamless three-dimensional carbon nanotube graphene hybrid material. *Nature communications* **2012,** *3* (1), 1225.

6. Wang, W.; Ruiz, I.; Guo, S.; Favors, Z.; Bay, H. H.; Ozkan, M.; Ozkan, C. S., Hybrid carbon nanotube and graphene nanostructures for lithium ion battery anodes. *Nano Energy* **2014,** *3*, 113-118.
